# Characterization of Hippocampal-Thalamic-Cortical Morphometric Reorganization in Temporal Lobe Epilepsy

**DOI:** 10.3389/fneur.2021.810186

**Published:** 2022-02-10

**Authors:** Hsin Tung, Szu-Yen Pan, Tsuo-Hung Lan, Yung-Yang Lin, Syu-Jyun Peng

**Affiliations:** ^1^Institute of Clinical Medicine, National Yang Ming Chiao Tung University, Taipei, Taiwan; ^2^Center of Faculty Development, Taichung Veterans General Hospital, Taichung, Taiwan; ^3^Division of Epilepsy, Neurological Institute, Taichung Veterans General Hospital, Taichung, Taiwan; ^4^Department of Neurosurgery, Neurological Institute, Taichung Veterans General Hospital, Taichung, Taiwan; ^5^Tsaotun Psychiatric Center, Ministry of Health and Welfare, Nantou, Taiwan; ^6^Department of Medicine, National Yang Ming Chiao Tung University, Taipei, Taiwan; ^7^Center for Neuropsychiatric Research, National Health Research Institutes, Zhunan, Taiwan; ^8^Institute of Clinical Medicine, National Yang Ming Chiao Tung University, Taipei, Taiwan; ^9^Department of Critical Care Medicine, Taipei Veterans General Hospital, Taipei, Taiwan; ^10^Institute of Brain Science, National Yang Ming Chiao Tung University, Taipei, Taiwan; ^11^Professional Master Program in Artificial Intelligence in Medicine, College of Medicine, Taipei Medical University, Taipei, Taiwan

**Keywords:** gray matter density, thalamus, anterior thalamic nucleus, hippocampal-thalamic-cortical network, temporal lobe epilepsy

## Abstract

**Introduction:**

Brain cortico-subcortical connectivity has been investigated in epilepsy using the functional MRI (MRI). Although structural images cannot demonstrate dynamic changes, they provide higher spatial resolution, which allows exploration of the organization of brain in greater detail.

**Methods:**

We used high-resolution brain MRI to study the hippocampal-thalamic-cortical networks in temporal lobe epilepsy (TLE) using a volume-based morphometric method. We enrolled 22 right-TLE, 33 left-TLE, and 28 age/gender-matched controls retrospectively. FreeSurfer software was used for the thalamus segmentation.

**Results:**

Among the 50 subfields, ipsilateral anterior, lateral, and parts of the intralaminar and medial nuclei, as well as the contralateral parts of lateral nuclei had significant volume loss in both TLE. The anteroventral nucleus was most vulnerable. Most thalamic subfields were susceptible to seizure burden, especially the left-TLE. SPM12 was used to conduct an analysis of the gray matter density (GMD) maps. Decreased extratemporal GMD occurred bilaterally. Both TLE demonstrated significant GMD loss over the ipsilateral inferior frontal gyrus, precentral gyrus, and medial orbital cortices.

**Significance:**

Thalamic subfield atrophy was related to the ipsilateral inferior frontal GMD changes, which presented positively in left-TLE and negatively in right-TLE. These findings suggest prefrontal-thalamo-hippocampal network disruption in TLE.

## Introduction

Focal epilepsy is considered as a network disorder, with extensive ipsilateral and contralateral involvement. Previously, only superficial cortices were thought to be parts of the epilepsy networks. However, there are growing evidences of the role of deep gray matters in epilepsy networks (1).

Basal ganglion and thalamus are the important gray matter located in the subcortical regions, and they extend many connections to the other cortical regions. Cortico-subcortical connectivity was reported to be related to the behavioral regulation (2) and cognitive control (3). The thalamus is an important subcortical hub in the brain, projecting fibers to nearly the whole cerebral cortex. It receives reciprocal connections from the cortices for processing and integration (4). The neuronal signals are delivered to the individual thalamic nuclei, involving somatosensory, motor, emotion/memory, and states of consciousness. Thalamic volume and the associated thalamo-cortical connectivity correspond to cognitive performances, motor task behaviors, and verbal memory (5).

The thalamus is divided into different parts, which are based on their specific connections consisting of different types of neurons. For example, anterior thalamic nucleus primarily connects with the cingulate gyrus and limbs system, involving alertness, attention, and memory (6). Most ventral parts of the lateral group, pulvinar, and medial dorsal nuclei have connections with the four cerebral lobes, processing somatic sensory and voluntary motor functions (6). The nuclei of the medial group connect with the prefrontal cortex and hypothalamus, taking part in emotion, thought, and judgment.

In addition, the thalamus participates in seizure maintenance, progression (7), and ictal conscious statement (8, 9). It presents epileptogenicity (10) in both the primarily and secondarily generalized tonic-clonic seizures (11). Disturbances of the cortico-subcortical network occurred in the focal epilepsy regardless of the localization (12). There is also evidence that epilepsy can alter the thalamo-cortical networks (13). Ipsilateral and contralateral thalamic involvement has been observed. This has been shown to be related to seizure severity in an animal kindling model (14), and was found to be a prognostic factor in epilepsy surgery (15).

Alteration of the thalamus in temporal lobe epilepsy (TLE) has been reported in previous studies of structural (16), metabolic (17–19), and functional (20) aspects of the thalamus. These studies demonstrated that TLE affects the hippocampo-thalamic network. Structurally, bilateral thalami and parietal lobes atrophy were found by conducting voxel-based morphometry (VBM) studies of magnetic resonance images (16, 21). However, the controversial result has been reported, which stated no significant changes in the thalamic volume (22). Using metabolic imaging, brain PET showed that signals of the ipsilateral thalamus decreased in the temporal and frontal lobe epilepsy (19). Resting-state functional MRI disclosed higher power in the ipsilateral thalamus than in the contralateral thalamus in TLE (20). Anterior, dorsomedial, and pulvinar nuclei were reported as the most preferentially affected thalamic subfields in TLE (7, 20, 23), which can be explained by the close anatomical connectivity profiles between the hippocampus and the thalamus.

Gray matter abnormalities have been described in epilepsy and other neurodegenerative disorders (24), suggesting regional neuronal injury and plasticity. Reconstruction of the cortical thickness of the brain cortical surface can be performed to study the neuropathological heterogenicity. It is the most common method applied in the epilepsy field, as it allows identification of extensive thinning of the extratemporal cortices in TLE (21, 25, 26). Gray matter density (GMD) calculates the regional proton density to reflect the brain tissue microenvironment (27), which has been used in investigations of epilepsy (13, 28). Both analytic techniques employ voxel-based morphometry (VBM) to quantify the neuronal volumes. However, their analytic approaches differ, and the results are not completely unanimous. Compared with the cortical thickness, GMD was shown to be more sensitive in the detection of gray matter loss and CSF volume increase (29).

Both hippocampo-thalamic and thalamo-cortical network alterations in epilepsy have been well studied individually, mostly by the functional imaging, such as functional MRI (fMRI). However, detailed information on how the hippocampus, the thalamus, and the cortex reorganize their connections in TLE have not been well identified due to the limited spatial resolution. Therefore, we used imaging with higher spatial resolution structurally to identify the topological changes of the hippocampo-thalamo-cortical volumetric reorganization in TLE.

## Methods

### Subjects

This was an observational study, and we collected the epilepsy patients from Taichung Veterans General Hospital (TCVGH) between June 2020 and December 2020 retrospectively. The enrolled cases had pharmaco-resistant TLE, and the epileptogenic zone was confirmed to be located at one side of the medial temporal after comprehensive survey, with or without the involvement of part of the lateral temporal cortex. After serial exams, such as epilepsy histories, clinical semiology, video-electroencephalography (vEEG), high resolution brain MRI, and PET imaging, TLE was diagnosed. Besides, their epilepsy duration, seizure frequency, and the presence of interictal discharges (IEDs) on the 20-min routine EEG exam were recorded, and these three parameters were deemed to reflect the seizure burden. Epilepsy duration was defined as the number of years from diagnosis of epilepsy to 2020. Seizure frequency was defined as the average number of detected seizure events in 6 months based on their medical records in 2020. The presence of the IEDs was determined based on their EEG exam in 2020, which qualitatively revealed the frequency of IEDs. The yes or no results were confirmed by a neurologist and a neurosurgeon. The healthy controls were recruited from the families of the epilepsy patients, whose health status was relatively normal and did not have epilepsy. We excluded subjects who had any of the following: brain tumor, history of head trauma or stroke, and severe psychiatric disorders, such as depression. This study was approved by the Ethics Committee of Taichung Veterans General Hospital, Taichung, Taiwan (CE18306B).

### MRI Protocol

All enrolled patients underwent MRI at the TCVGH with a 1.5-T scanner (Aera, Siemens, Erlangen, Germany), using a 20-channel phase-array head coil. The T1-weighted 3-dimensional magnetization-prepared rapid acquisition with gradient-echo (3D-MPRAGE) images were obtained using the standard parameters. This includes the following: repetition time = 2,800 ms, inversion time = 930 ms, echo time = 5.13 ms, spatial resolution = 0.8 × 0.8 × 1.0 mm^3^, flip angle = 8°. Patients with poor MRI quality, such as motion or misalignment artifacts, were excluded.

### Volumetric Analysis for Division of the Thalamic Subfields

For each subject, we carried out an ROI-based analysis, with automated segmentation and quantitation of the thalamic subfields from their 3D-MPRAGE images using FreeSurfer version 7.1.1. (https://surfer.nmr.mgh.harvard.edu/fswiki/FreeSurferWiki). The standard FreeSurfer “recon-all” processing pipeline was used for anatomical parcellation. Automatic labeling and volumetric quantification of thalamic subfields were conducted using the segmentation of the individual whole thalamic nuclei, and then the adaptive segmentation technique was performed (30). There are 25 subfields on each side of the thalamus, such as anteroventral (AV), laterodorsal (LD), lateral posterior (LP), ventral anterior (VA), ventral anterior magnocellular (Vamc), ventral lateral anterior (VLa), ventral lateral posterior (VLp), ventral posterolateral (VPL), ventromedial (VM), central medial (CeM), central lateral (CL), paracentral (Pc), centromedian (CM), parafascicular (Pf), paratenial (Pt), reuniens-medial ventral [MV(Re)], mediodorsal medial magnocellular (MDm), mediodorsal lateral parvocellular (MDl), lateral geniculate (LGN), medial geniculate (MGN), limitans-suprageniculate (L-Sg), pulvinar anterior (PuA), pulvinar medial (PuM), pulvinar lateral (PuL), and pulvinar inferior (PuI). Individual thalamic subfields and the whole thalamus nuclei on both sides were divided by the estimated total intracranial volume (eTIV) for volume normalization.

### Morphometric Analysis of GMD

The 3D-MPRAGE images were reoriented based on the origin of the anterior commissure, and then segmented into gray matter, white matter, and cerebrospinal fluid using the segment module of the SPM12 software (31). SPM12 was used to conduct the voxel-based morphometric analysis (http://www.fil.ion.ucl.ac.uk/spm/) with MATLAB R2021a (Mathworks, Natick, MA, USA). The GMD maps were non-linearly transferred to a Montreal Neurological Institute (MNI) space using a Diffeomorphic Anatomical Registration Through Exponentiated Lie Algebra (DARTEL) algorithm (32) and transformed to 1 × 1 × 1 mm cubic voxel size. To avoid focal deformation caused by non-linear transformations, such as excessive compression and expansion, we modulated the GMD maps using the Jacobian determinants of the deformation module of SPM12 (33). Finally, the modulated GMD maps were spatially smoothed with a Gaussian kernel with full width at the half maximum of 8 mm. Afterward, we established the relationship of volumetric changes between the cortical gray matter (GMD) and the deep structure (thalamus) in L-TLE and R-TLE. Thus, we selected the thalamic subfields whose volumes were significantly decreased in each TLE based on the method described in Section Volumetric Analysis for Division of the Thalamic Subfields. These subfields on each side were treated as a single unit, resulting in four thalamic units (left hemispheric and right hemispheric in both TLE). Then, we used correlation analysis to identify the associated cortical areas, whose GMD changes had the same degree of volumetric changes as these four thalamic units. In addition, the analysis was also adjusted for age and gender.

We had performed a subgroup analysis for hippocampal sclerosis in the right-TLE and left-TLE groups. However, only 8 in 22 cases (36.4%) and 16 in 33 cases (48.5%) had hippocampal sclerosis in the right-TLE and left-TLE groups, respectively. The 28 controls became poorly comparable with the selected hippocampal sclerotic cases, and the results did not show obvious significance of the volumes of thalamic subfields. Therefore, we did not show this result of subgroup analysis.

### Statistical Analysis

The differences in the individual normalized volumes of the thalamic subfields were analyzed by multivariate analysis of covariance (MANCOVA) among the left-TLE (L-TLE), right-TLE (R-TLE), and control groups. The results were adjusted for age and gender, and then Bonferroni correction was applied for multiple comparison correction. To establish the relationship between the thalamic subfield volume and the epilepsy duration, seizure frequency, and seizure burden, Spearman's partial correlation was used with adjustment for age and gender. When the adjusted values of *p* were less than 0.05, they were considered to be statistically significant. SPSS version 18.0 (IBM, SPSS Inc., Chicago, IL, USA) was used for all statistical analyses.

For group comparison of GMD, a two-sample *t*-test was used with adjustment for gender and age. Although family-wise error rate (FWER) or false discovery rate (FDR) is the default analytic method of SPM12, but it exerts a more stringent control. Due to our limited sample size, the AlphaSim was applied for the correction of multiple comparisons. The AlphaSim provided a means of evaluating the probability of a false detection within the gray matter mask and is performed by Monte Carlo simulation (34). The probability of a false positive detection is determined from the frequency count of cluster sizes based on the combination of individual voxel probability thresholding and minimum cluster size thresholding. The significance levels were set when *p* < 0.01 with a minimum cluster size of 354 voxels (34).

## Results

There were 23 R-TLE, 32 L-TLE, and 28 age- and gender-matched controls collected. The characteristics of the three groups, such as age, gender, and eTIV, are listed in Table 1, and no significant differences in these variables were noted. The three parameters reflecting seizure burden were similar between the L-TLE and the R-TLE groups. The kinds of AED used were similar.

**Table 1:** Demographic characteristics of control, R-TLE, and L-TLE groups.

	**Control (n = 28)**	**R-TLE (n = 23)**	***P* ^&^**	**L-TLE (n = 32)**	***P* ^%^**	***P* ^†^**
Sex			1.000^$^		0.390^$^	0.420^$^
Male	13(46.4%)	11 (47.8%)		20(62.5%)		–
Female	15(53.6%)	12 (52.2%)		12 (37.5%)		–
Age	47.50(31.25–57.75)	39.00 (29.00–54.00)	0.733	34.50 (29.50–50.75)	0.172	0.398
Seizure duration (years)	NA	9.00 (2.00–25.00)	–	9.50 (2.00–20.75)	–	0.905
Seizure frequency (each month)	NA	2.00 (0.50–3.00)	–	2.00 (0.50–3.00)	–	0.478
Spike on the routine EEG	NA	10.00 (43.5%)	–	18.00 (56.3%)	–	0.509^$^
eTIV	1458512.89 (1,377,721.75–1629270.98)	1449314.09 (1,336,183.00–1543227.60)	0.364	1466867.67 (1,393,644.93–1651023.27)	0.583	0.210

*Mann-Whitney U test*:

& *comparing R-TLE and control*,

% *comparing L-TLE and control*,

† *comparing R-TLE and L-TLE*.

$ *Chi-square test*.

*Median (IQR), eTIV, estimated total intracranial volume*.

### Ipsilateral Anteroventral Nucleus Exhibited the Most Atrophy

**Figure 1:**
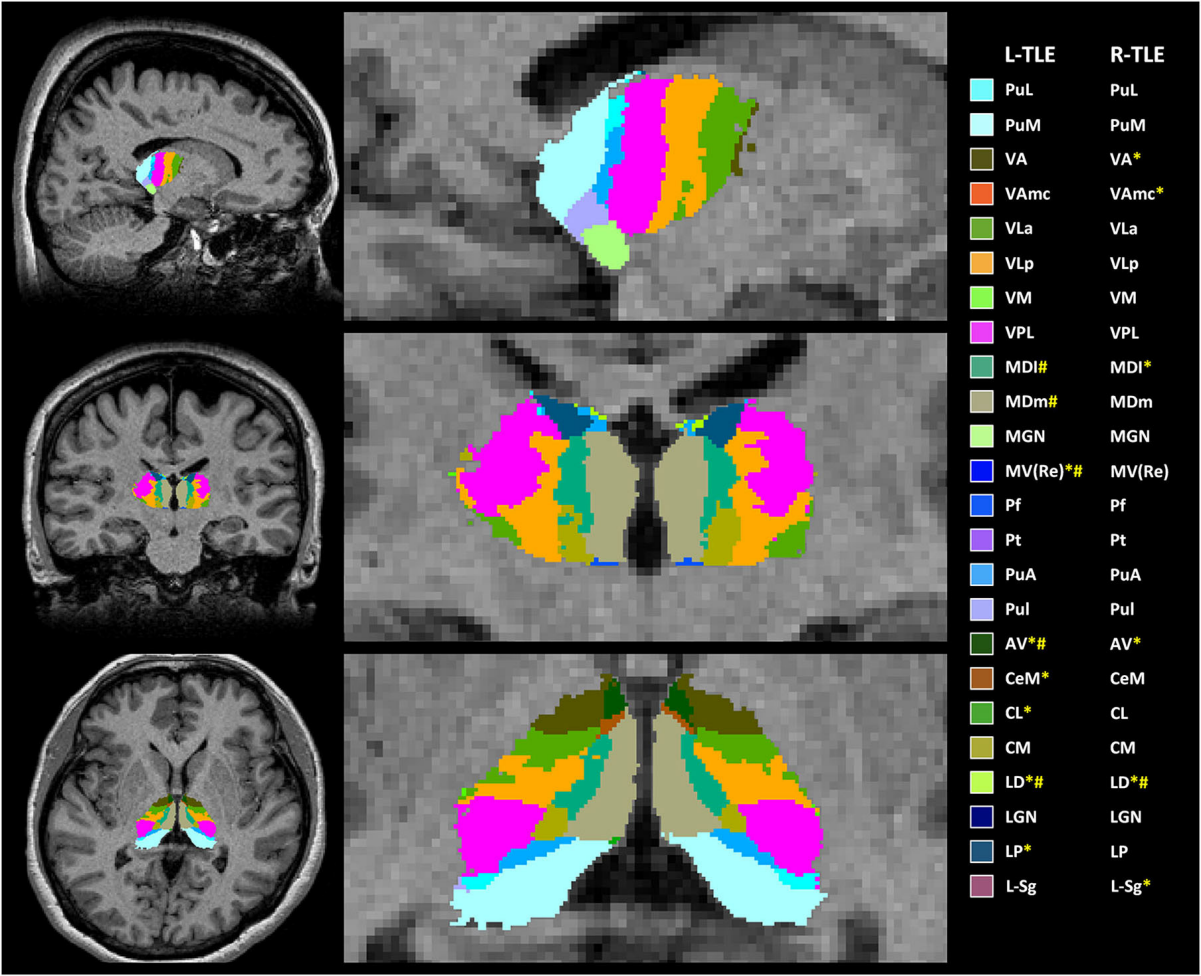
Example of anatomical locations of the thalamic subfields generated by FreeSurfer on 3-dimensional magnetization-prepared rapid acquisition with gradient-echo (3D-MPRAGE) images in one of the patients with left-temporal lobe epilepsy (L-TLE) (not all segmentations are shown). ^*^ TLE had significantly decreased volume of thalamic subfields in the right hemisphere; # TLE had significantly decreased volume of thalamic subfields in the left hemisphere. AV, anteroventral; LD, laterodorsal; LP, lateral posterior; VA, ventral anterior; Vamc, ventral anterior magnocellular; VLa, ventral lateral anterior; VLp, ventral lateral posterior; VPL, ventral posterolateral; VM, ventromedial; CeM, central medial; CL, central lateral; Pc, paracentral; CM, centromedian; Pf, parafascicular; Pt, paratenial; MV(Re), reuniens (medial ventral); MDm, mediodorsal medial magnocellular; MDl, mediodorsal lateral parvocellular; LGN, lateral geniculate; MGN, medial Geniculate; L-Sg, limitans (suprageniculate); PuA, pulvinar anterior; PuM, pulvinar medial; PuL, pulvinar lateral; PuI, pulvinar inferior.

The normalized volume of both sides of the whole thalami in both TLE patient groups seemed to be similar to that of the controls. When the thalamus was divided into 25 thalamic subfields on each side (Figure 1), significant volume loss was observed in several subfields, which was more prominent on the ipsilateral side in both TLE groups. Compared with controls, both TLE patient groups had significantly decreased volume over the ipsilateral anterior (AV), lateral (LD, LP), and parts of intralaminar (CeM) and medial [MV(Re)] nuclei, as well as the contralateral parts of lateral nuclei (Table 2). Among these structures, the ipsilateral anteroventral nucleus had the most significant volume loss (L-TLE: *p* < 0.001, R-TLE: *p* = 0.003), especially L-TLE. In contrast, R-TLE had more extensive volume reduction of the ipsilateral thalamic nuclei, extending to the posterior subnuclei. Laterodorsal nucleus was the only nucleus showing significant bilateral volume decrease in each TLE group.

**Table 2:** The volume of each thalamic nuclei in the two pharmaco-resistant epilepsy groups and the controls.

		**Control**		**L-TLE**			**R-TLE**		
**Groups**		**Mean^a^**	**Standard error**	**Mean^a^**	**Standard error**	**p^b^**	**Mean^a^**	**Standard error**	**p^b^**
L-Anterior	L-AV	1.042	0.026	0.903	0.025	<0.001^*^	0.950	0.029	0.062
L-Lateral	L-LD	0.243	0.012	0.179	0.012	0.001^*^	0.190	0.014	0.019^*^
	L-LP	0.955	0.025	0.861	0.024	0.027^*^	0.868	0.028	0.072
L-Ventral	L-VA	3.002	0.054	2.843	0.052	0.117	2.837	0.062	0.144
	L-Vamc	0.234	0.005	0.223	0.005	0.349	0.221	0.005	0.198
	L-VLa	4.376	0.085	4.217	0.082	0.548	4.221	0.097	0.690
	L-VLp	5.703	0.119	5.470	0.115	0.502	5.502	0.136	0.807
	L-VPL	6.079	0.146	5.951	0.141	1.000	5.799	0.167	0.625
	L-VM	0.179	0.006	0.167	0.006	0.452	0.162	0.007	0.178
L-Intralaminar	L-CeM	0.518	0.013	0.468	0.013	0.025^*^	0.475	0.015	0.104
	L-CL	0.310	0.012	0.258	0.011	0.007^*^	0.266	0.013	0.041^*^
	L-Pc	0.027	0.001	0.025	0.001	0.080	0.025	0.001	0.133
	L-CM	1.727	0.037	1.704	0.035	1.000	1.669	0.042	0.885
	L-Pf	0.388	0.010	0.387	0.010	1.000	0.384	0.012	1.000
L-Medial	L-Pt	0.050	0.001	0.048	0.001	0.847	0.047	0.001	0.440
	L-MV(Re)	0.096	0.003	0.084	0.003	0.019^*^	0.085	0.004	0.053
	L-MDm	5.078	0.104	4.794	0.100	0.166	4.956	0.119	1.000
	L-MDl	1.823	0.035	1.743	0.034	0.316	1.724	0.040	0.198
L-Posterior	L-LGN	1.800	0.042	1.696	0.041	0.239	1.698	0.048	0.341
	L-MGN	0.790	0.018	0.776	0.018	1.000	0.731	0.021	0.117
	L-L-Sg	0.189	0.007	0.194	0.006	1.000	0.171	0.008	0.275
	L-PuA	1.405	0.025	1.339	0.024	0.192	1.371	0.028	1.000
	L-PuM	7.454	0.148	7.104	0.142	0.284	7.252	0.169	1.000
	L-PuL	1.273	0.033	1.272	0.032	1.000	1.203	0.038	0.490
	L-PuI	1.656	0.037	1.632	0.035	1.000	1.640	0.042	1.000
	L-whole thalamus^c^	46.396	0.809	44.340	0.781	0.220	44.447	0.926	0.348
R-Anterior	R-AV	1.069	0.024	0.989	0.023	0.060	0.945	0.028	0.003^*^
R-Lateral	R-LD	0.242	0.012	0.194	0.012	0.016^*^	0.186	0.014	0.008^*^
	R-LP	0.926	0.023	0.860	0.022	0.129	0.835	0.026	0.030^*^
R-Ventral	R-VA	2.903	0.050	2.792	0.048	0.348	2.661	0.057	0.006^*^
	R-VAmc	0.239	0.004	0.230	0.004	0.409	0.222	0.005	0.029^*^
	R-VLa	4.393	0.077	4.258	0.075	0.654	4.138	0.089	0.099
	R-VLp	5.724	0.109	5.553	0.105	0.799	5.449	0.125	0.299
	R-VPL	6.304	0.160	6.153	0.154	1.000	5.979	0.183	0.550
	R-VM	0.188	0.006	0.178	0.006	0.792	0.172	0.007	0.326
R-Intralaminar	R-CeM	0.524	0.014	0.488	0.014	0.229	0.469	0.016	0.039^*^
	R-CL	0.314	0.012	0.277	0.012	0.110	0.269	0.014	0.062
	R-Pc	0.030	0.001	0.029	0.001	0.182	0.028	0.001	0.102
	R-CM	1.763	0.037	1.706	0.035	0.804	1.653	0.042	0.151
	R-Pf	0.415	0.010	0.403	0.010	1.000	0.393	0.011	0.476
R-Medial	R-Pt	0.055	0.001	0.052	0.001	0.208	0.051	0.001	0.075
	R-MV(Re)	0.102	0.004	0.088	0.004	0.029^*^	0.087	0.004	0.035^*^
	R-MDm	5.216	0.100	4.893	0.096	0.069	4.983	0.114	0.377
	R-MDl	1.910	0.034	1.797	0.032	0.059	1.761	0.039	0.014^*^
R-Posterior	R-LGN	1.874	0.051	1.823	0.050	1.000	1.748	0.059	0.327
	R-MGN	0.880	0.022	0.877	0.021	1.000	0.836	0.025	0.551
	R-L-Sg	0.179	0.006	0.170	0.006	0.918	0.148	0.007	0.004^*^
	R-PuA	1.477	0.028	1.422	0.027	0.491	1.401	0.032	0.233
	R-PuM	7.940	0.164	7.796	0.159	1.000	7.564	0.188	0.403
	R-PuL	1.290	0.034	1.331	0.033	1.000	1.250	0.039	1.000
	R-PuI	1.796	0.040	1.785	0.039	1.000	1.721	0.046	0.644
	R-whole thalamus^c^	47.755	0.834	46.144	0.805	0.516	44.951	0.955	0.088

a *The values were the ratio of estimated total intracranial volume (eTIV), which were then corrected by multiplying by 10^5^*.

b *Comparison with the controls using Multivariate Analysis of Covariance (MANCOVA), with age and gender as the covariates, followed by adjustment by Bonferroni correction (using the following values to estimate the covariates that appear in the model: age = 41.33)*.

c *Comparison with the controls using Analysis of Covariance (ANCOVA), with age and gender as the covariates, followed by adjustment by Bonferroni correction*.

* *p < 0.05, which was presented with bold values*.

*L, Left; R, right; TLE, temporal lobe epilepsy*.

*AV, Anteroventral; LD, Laterodorsal; LP, Lateral posterior; VA, Ventral anterior; Vamc, Ventral anterior magnocellular; VLa, Ventral lateral anterior; VLp, Ventral lateral posterior; VPL, Ventral posterolateral; VM, Ventromedial; CeM, Central medial; CL, Central lateral; Pc, Paracentral; CM, Centromedian; Pf, Parafascicular; Pt, Paratenial; MV(Re), Reuniens (medial ventral); MDm, Mediodorsal medial magnocellular; MDl, Mediodorsal lateral parvocellular; LGN, Lateral geniculate; MGN, Medial Geniculate; L-Sg, Limitans (suprageniculate); PuA, Pulvinar anterior; PuM, Pulvinar medial; PuL, Pulvinar lateral; PuI, Pulvinar inferior*.

### Seizure Burden Parameters Were Related to the Thalamic Subfield Volumes

In our study, the seizure burden was reflected by transient (spike on the routine EEG exam), moderate (seizure frequency in recent 6 months), and remote (epilepsy duration) effects. They were related to several thalamic subfield volumes, except for spikes on the routine EEG in R-TLE (Table 3). In L-TLE, most thalamic subfields on the left side were negatively correlated with epilepsy duration, especially the anterior, lateral ventral, intralaminar, medial, and posterior nuclei, as well as the whole left thalamus. Suprageniculate nucleus was the only one exception among the left thalamic nuclei. In R-TLE, the number of thalamic subfields related to epilepsy duration was lower. Seizure frequency in L-TLE was not related to any ipsilateral thalamic nuclei, but was positively correlated with the three right thalamic nuclei (ventral lateral posterior, parafascicular, and suprageniculate nuclei). In contrast, seizure frequency in R-TLE was negatively correlated with the left anterior, ventral, and interlaminar nuclei, and only one ipsilateral central lateral nucleus. Our routine EEG was only performed for 20 min in the daytime, as the patient was almost awake. If any spikes were detected in the routine EEG, this suggested that the spikes occurred relatively frequently. This condition in L-TLE was correlated with widespread volume loss of the bilateral thalamic subfields.

**Table 3:** The relationship between hippocampus subfield volumes and the clinical characteristics.

		**L-TLE**						**R-TLE**					
		**Seizure duration**		**Seizure frequency**		**Spikes in routine EEG**		**Seizure duration**		**Seizure frequency**		**Spikes in routine EEG**	
		**R**	**p**	**R**	**p**	**R**	**p**	**R**	**p**	**R**	**p**	**R**	**p**
L-Anterior	L-AV	−0.590	<0.001^*^	0.050	0.789	−0.327	0.072	−0.542	0.014^*^	−0.562	0.010^*^	−0.264	0.261
L-Lateral	L-LD	−0.444	0.012^*^	−0.022	0.905	−0.176	0.343	−0.402	0.079	−0.419	0.066	−0.140	0.556
	L-LP	−0.532	0.002^*^	0.028	0.883	−0.191	0.304	−0.153	0.519	−0.264	0.261	−0.024	0.919
L-Ventral	L-VA	−0.608	<0.001^*^	0.156	0.402	−0.397	0.027^*^	−0.356	0.124	−0.452	0.045^*^	−0.016	0.946
	L-Vamc	−0.607	<0.001^*^	0.205	0.269	−0.407	0.023^*^	−0.328	0.158	−0.337	0.146	0.032	0.893
	L-VLa	−0.582	0.001^*^	0.219	0.237	−0.342	0.060	−0.280	0.231	−0.386	0.093	0.010	0.967
	L-VLp	−0.578	0.001^*^	0.201	0.279	−0.345	0.057	−0.279	0.234	−0.315	0.176	0.043	0.857
	L-VPL	−0.554	0.001^*^	0.115	0.539	−0.358	0.048^*^	−0.281	0.231	−0.216	0.360	0.103	0.666
	L-VM	−0.456	0.010^*^	0.172	0.354	−0.321	0.078	−0.257	0.275	−0.210	0.375	0.053	0.825
L-Intralaminar	L-CeM	−0.593	<0.001^*^	0.139	0.456	−0.397	0.027^*^	−0.314	0.177	−0.366	0.113	−0.229	0.332
	L-CL	−0.385	0.032^*^	0.083	0.658	−0.256	0.164	−0.414	0.070	−0.535	0.015^*^	−0.268	0.254
	L-Pc	−0.614	<0.001^*^	0.228	0.217	−0.367	0.042^*^	−0.364	0.115	−0.506	0.023^*^	−0.275	0.241
	L-CM	−0.555	0.001^*^	0.224	0.225	−0.233	0.208	−0.266	0.257	−0.347	0.134	−0.020	0.933
	L-Pf	−0.457	0.010^*^	0.253	0.170	−0.146	0.433	−0.166	0.483	−0.301	0.197	−0.070	0.770
L-Medial	L-Pt	−0.549	0.001^*^	0.129	0.488	−0.397	0.027^*^	−0.501	0.024^*^	−0.261	0.266	−0.106	0.656
	L-MV(Re)	−0.473	0.007^*^	0.123	0.509	−0.486	0.006^*^	−0.437	0.054	−0.217	0.358	−0.068	0.774
	L-MDm	−0.420	0.019^*^	0.042	0.823	−0.364	0.044^*^	−0.147	0.535	−0.278	0.235	−0.056	0.813
	L-MDl	−0.414	0.021^*^	0.013	0.943	−0.289	0.115	−0.338	0.144	−0.371	0.108	−0.106	0.655
L-Posterior	L-LGN	−0.429	0.016^*^	−0.039	0.835	−0.277	0.132	−0.097	0.686	−0.131	0.583	−0.241	0.307
	L-MGN	−0.486	0.006^*^	0.159	0.392	−0.339	0.062	−0.460	0.041^*^	−0.373	0.105	−0.350	0.131
	L-L-Sg	−0.278	**0.130**	0.290	0.113	0.034	0.857	−0.159	0.503	−0.181	0.446	−0.310	0.184
	L-PuA	−0.594	<0.001^*^	0.006	0.975	−0.301	0.099	0.226	0.337	0.099	0.679	0.189	0.425
	L-PuM	−0.451	0.011^*^	−0.052	0.781	−0.190	0.305	0.127	0.594	−0.052	0.827	−0.031	0.897
	L-PuL	−0.607	<0.001^*^	0.014	0.941	−0.368	0.041^*^	−0.090	0.705	−0.129	0.589	−0.053	0.825
	L-PuI	−0.426	0.017^*^	−0.070	0.709	−0.257	0.164	0.129	0.588	0.104	0.663	−0.002	0.994
	L-whole thalamus	−0.624	<0.001^*^	0.105	0.575	−0.369	0.041^*^	−0.216	0.361	−0.297	0.204	−0.039	0.872
R-Anterior	R-AV	−0.262	0.154	−0.044	0.813	−0.388	0.031^*^	−0.453	0.045^*^	−0.332	0.153	−0.311	0.181
R-Lateral	R-LD	−0.270	0.142	0.096	0.608	−0.384	0.033^*^	−0.507	0.023^*^	−0.337	0.146	0.124	0.602
	R-LP	−0.323	0.076	0.194	0.296	−0.243	0.187	−0.367	0.111	−0.417	0.068	0.115	0.628
R-Ventral	R-VA	−0.339	0.062	0.160	0.389	−0.301	0.100	−0.343	0.139	−0.308	0.187	−0.163	0.492
	R-VAmc	−0.382	0.034^*^	0.209	0.260	−0.272	0.138	−0.334	0.150	−0.314	0.178	−0.105	0.658
	R-VLa	−0.351	0.053	0.350	0.054	−0.187	0.313	−0.267	0.255	−0.326	0.161	−0.074	0.757
	R-VLp	−0.348	0.055	0.363	0.044^*^	−0.213	0.249	−0.238	0.312	−0.255	0.277	0.030	0.901
	R-VPL	−0.358	0.048^*^	0.350	0.053	−0.156	0.401	−0.135	0.570	−0.277	0.237	0.061	0.799
	R-VM	−0.377	0.037^*^	0.350	0.054	−0.194	0.296	−0.164	0.490	−0.311	0.182	0.052	0.828
R-Intralaminar	R-CeM	−0.412	0.021^*^	0.084	0.654	−0.361	0.046^*^	−0.392	0.087	−0.311	0.182	−0.255	0.277
	R-CL	−0.105	0.575	0.254	0.168	−0.371	0.040^*^	−0.510	0.022^*^	−0.500	0.025^*^	−0.034	0.886
	R-Pc	−0.350	0.053	0.242	0.189	−0.263	0.153	−0.277	0.237	−0.229	0.331	−0.243	0.301
	R-CM	−0.406	0.023^*^	0.333	0.067	−0.121	0.516	−0.182	0.443	−0.215	0.362	0.110	0.645
	R-Pf	−0.437	0.014^*^	0.399	0.026^*^	−0.079	0.674	−0.104	0.661	−0.178	0.452	0.011	0.963
R-Medial	R-Pt	−0.456	0.010^*^	0.257	0.163	−0.349	0.054	−0.380	0.098	−0.342	0.140	0.000	0.999
	R-MV(Re)	−0.370	0.041^*^	0.046	0.806	−0.361	0.046^*^	−0.387	0.092	−0.281	0.230	−0.134	0.572
	R-MDm	−0.188	0.312	0.086	0.644	−0.285	0.121	−0.209	0.377	−0.353	0.127	0.061	0.799
	R-MDl	−0.278	0.130	0.074	0.692	−0.395	0.028^*^	−0.212	0.370	−0.307	0.188	0.022	0.927
R-Posterior	R-LGN	−0.067	0.722	0.174	0.349	−0.351	0.053	−0.109	0.647	−0.207	0.381	−0.019	0.936
	R-MGN	−0.399	0.026^*^	0.129	0.488	−0.373	0.039^*^	−0.501	0.025^*^	−0.423	0.063	−0.124	0.601
	R-L-Sg	−0.262	0.155	0.386	0.032^*^	−0.034	0.854	−0.514	0.020^*^	−0.393	0.086	−0.160	0.500
	R-PuA	−0.373	0.039^*^	0.173	0.351	−0.223	0.227	−0.110	0.644	−0.223	0.344	0.175	0.461
	R-PuM	−0.355	0.050^*^	0.148	0.426	−0.217	0.242	−0.171	0.471	−0.263	0.263	0.103	0.665
	R-PuL	−0.386	0.032^*^	0.169	0.363	−0.060	0.749	−0.035	0.884	−0.156	0.512	−0.188	0.427
	R-PuI	−0.255	0.166	0.149	0.425	−0.204	0.271	−0.067	0.781	−0.252	0.284	−0.032	0.892
	R-whole thalamus	−0.401	0.025^*^	0.283	0.123	−0.287	0.118	−0.249	0.290	−0.333	0.151	0.011	0.963

*R, partial correlation coefficient*.

* *p < 0.05, which was presented with bold values*.

*L, Left; R, Right*.

*AV, Anteroventral; LD, Laterodorsal; LP, Lateral posterior; VA, Ventral anterior; Vamc, Ventral anterior magnocellular; VLa, Ventral lateral anterior; VLp, Ventral lateral posterior; VPL, Ventral posterolateral; VM, Ventromedial; CeM, Central medial; CL, Central lateral; Pc, Paracentral; CM, Centromedian; Pf, Parafascicular; Pt, Paratenial; MV(Re), Reuniens (medial ventral); MDm, Mediodorsal medial magnocellular; MDl, Mediodorsal lateral parvocellular; LGN, Lateral geniculate; MGN, Medial Geniculate; L-Sg, Limitans (suprageniculate); PuA, Pulvinar anterior; PuM, Pulvinar medial; PuL, Pulvinar lateral; PuI, Pulvinar inferior*.

### The Extratemporal GMD Decreased in Both TLE, Primarily Involving Parts of the Frontal Lobe

**Figure 2:**
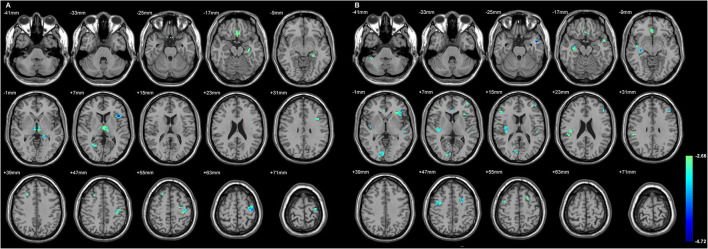
The cortices with significant gray matter density (GMD) changes compared with the normal controls, using age and gender as the covariates **(A)** in L-TLE, and **(B)** in right-LTE (R-TLE). All involved regions in both TLE showed decreased GMD, mostly located in the frontal and temporal lobes. Ipsilateral inferior frontal gyrus, precentral gyrus, and medial orbital cortices were affected in both TLE. A *T*-test comparison was performed between the two groups, using a double statistical threshold (height threshold *p* < 0.01 and a minimum cluster size = 354 voxels, as determined by the AlphaSim correction).

**Figure 3:**
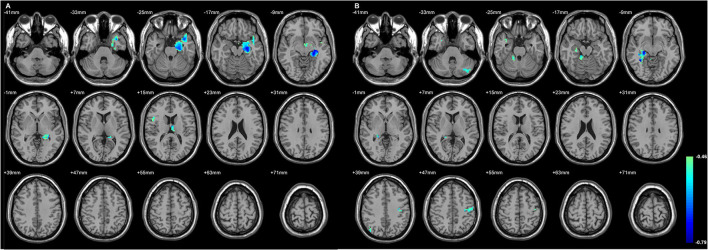
The regions with their GMD related to the epilepsy duration. The longer duration was related to decreased GMD in both TLE. **(A)** The involved cortices in L-TLE were in the left temporal and right inferior frontal areas. **(B)** The involved cortices in R-TLE were in the right temporal and left cerebellar areas. Differences with a *p* < 0.01 were considered significant (with a threshold *p* < 0.01 and a minimum cluster size = 354 voxels, as determined by the AlphaSim correction).

Figure 2 and Table 4 showed the significant GMD changes in both TLE groups compared with the controls. The commonly affected regions were the ipsilateral inferior frontal gyrus, precentral gyrus, and medial orbital cortex. Moreover, both TLE groups had significant GMD decreases over the left middle frontal gyrus and around the right calcarine areas. The cortex and its associations of GMD with epilepsy duration are listed in Table 5 and Figure 3.

**Table 4:** Group comparison of whole brain gray matter density (GMD) between the TLEs and the controls.

**Regions**	**MNI coordinate**	**Peak *t*-score**	**Number of voxels**
**(A) L-TLE vs. control**			
L Gyrus rectus	−3, 21, −27	−3.28	31
L Middle frontal gyrus	−39, 12, 36	−3.40	23
L Inferior frontal gyrus, triangular part	−39, 18, 6	−4.26	37
L Precentral gyrus	−33, −15, 66	−4.32	145
L Hippocampus	−18, −36, 3	−3.85	55
R Middle frontal gyrus	27, 24, 42	−3.45	31
R Calcarine fissure and surrounding cortex	27, −60, 6	−3.22	23
R Thalamus	9, −15, 0	−3.68	85
**(B) R-TLE vs. control**			
L Inferior frontal gyrus, triangular part	−42, 30, 0	−3.84	73
L Middle frontal gyrus	−48, 33, 30	−3.85	24
	−42, 51, 12	−4.34	28
	−24, 6, 45	−3.75	31
L Middle temporal gyrus	−48, −24, 0	−3.39	28
	−48, 3, −24	−4.72	32
R Inferior frontal gyrus, triangular part	48, 30, 9	−3.61	21
R Precentral gyrus	39, −6, 48	−3.83	41
R Olfactory cortex	3, 24, −3	−3.28	74
R Insula	33, −18, 18	−3.85	169
R Calcarine fissure and surrounding cortex	12, −87, 3	−4.00	53
R Hippocampus	33, −21, −9	−3.92	51
R Crus II of cerebellar hemisphere	39, −42, −42	−3.46	27

*The significantly different areas are listed below, and are also depicted in Figure 2. The values given are the stereotactic MNI coordinates and the t-score of each anatomical region. SPM maps used a threshold of p < 0.01 (AlphaSim correction, voxel-level, minimum. cluster size threshold of 19 voxels). Age and gender as covariates. MNI, Montreal Neurological Institute. L, left; R, right*.

**Table 5:** The cortical regions with gray matter density that was significantly related to the seizure duration in (A) L-TLE and (B) R-TLE.

**Regions**	**MNI coordinate**	**Peak *t*-score**	**Number of voxels**
**(A) L-TLE vs. control**			
L Lobule VIII of cerebellar hemisphere	−24, −39, −51	−0.57	30
L Hippocampus	−27, −21, −12	−0.74	652
L Thalamus	−3, −9, 15	−0.61	23
R Inferior frontal gyrus, opercular part	48, 12, 18	−0.53	24
**(B) R-TLE vs. control**			
L Postcentral gyrus	−33, −21, 42	−0.71	78
L Crus I of cerebellar hemisphere	−42, −78, −30	−0.74	38
R Temporal pole: superior temporal gyrus	36, 9, −27	−0.62	19
R Fusiform gyrus	36, −48, −9	−0.77	132
R Angular gyrus	45, −66, 36	−0.73	32
R Lobule IV, V of cerebellar hemisphere	18, −39, −21	−0.65	47

*The significant areas listed below are also listed in Figure 3. The values given are the stereotactic MNI coordinates and the t-score of each anatomical region. SPM maps were thresholded at p < 0.01 (AlphaSim correction, voxel-level, minimum cluster size threshold of 19 voxels). Age and gender as covariates. MNI, Montreal Neurological Institute. L, left; R, right*.

### The Status of the Volume Loss of Both Sides of Thalamic Subfield Was Primarily Related to the Ipsilateral Inferior Frontal GMD Changes

**Figure 4:**
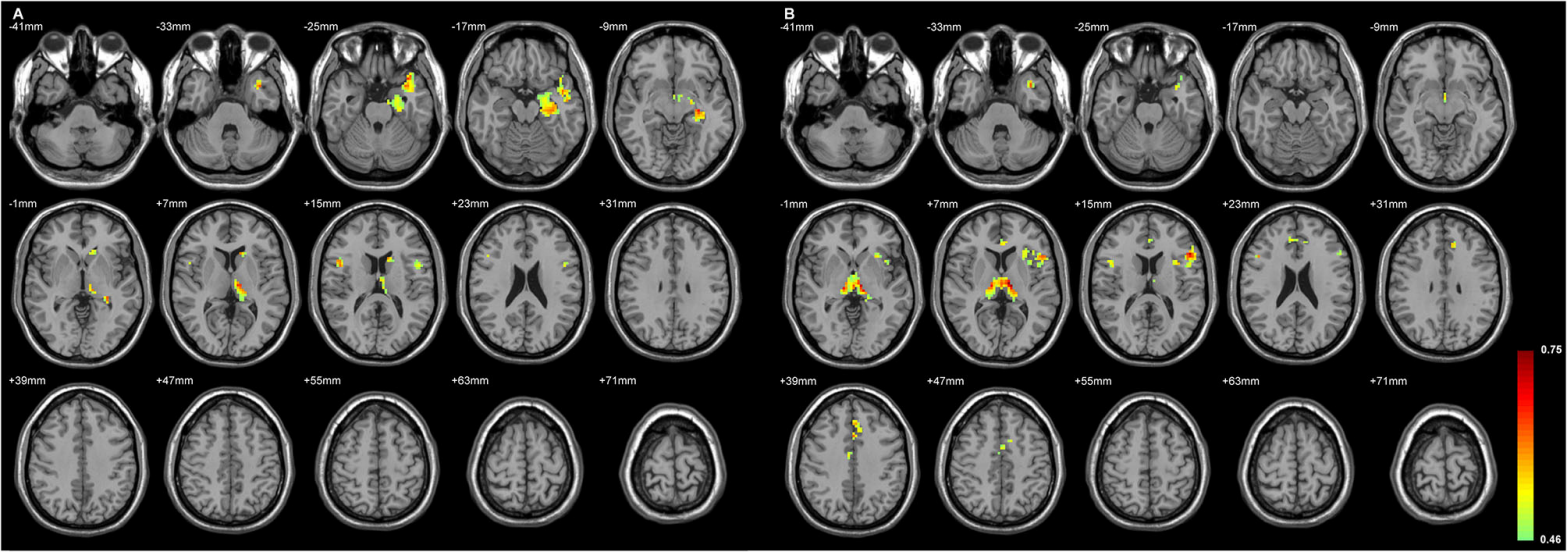
The regions with their GMD related to the **(A)** left AV-LD-LP- CeM-CL-MV(Re) volume loss and the **(B)** right LD-MV(Re) volume loss in L-TLE. All depicted regions showed a positive relationship, and were mostly located in the left frontal and temporal lobes. GMD of the left inferior frontal gyrus was positively related to each side of thalamic subfield volume changes in L-TLE. Differences with a *p* < 0.01 were considered significant (with a threshold *p* < 0.01 and a minimum cluster size = 354 voxels, as determined by the AlphaSim correction).

**Figure 5:**
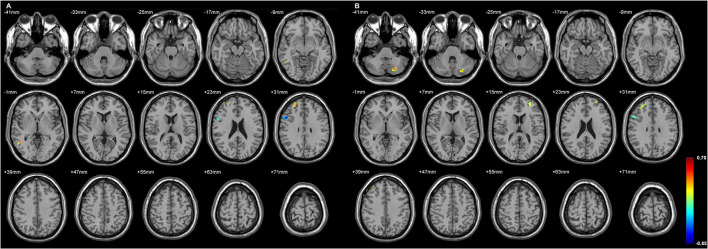
The regions with their GMD related to the **(A)** left LD-CL and the **(B)** right AV-LD-LP-VA-Vamc-CeM-MV(Re)-MDl-L-Sg volume changes in R-TLE. Both positive and negative relationships are depicted, and are mostly located in the right frontal lobe. GMD of the right inferior frontal gyrus was negatively related to each side of thalamic subfield volume changes in R-TLE. Differences with a *p* < 0.01 were considered significant (with a threshold *p* < 0.01 and a minimum cluster size = 354 voxels, as determined by the AlphaSim correction).

Figures 4, 5 exhibited that the distribution of the neocortical atrophy was strongly associated with the amount of the sum of volume loss of the thalamic subfields on each side. GMD of the ipsilateral inferior frontal gyrus was positively related to each side of thalamic subfield volume changes in L-TLE. Meanwhile, it was negatively correlated with the volume loss of each side of thalamic subfields in R-TLE. In addition to the thalamus, GMD of the left caudate nucleus was another deep structure that was related to the thalamic subfield volume changes in L-TLE (Table 6).

**Table 6:** The associated cortical area, whose GMD changes had the same degree of volumetric changes of the thalamic unit on each side in L-TLE.

**Regions**	**MNI coordinate**	**Peak *t*-score**	**Number of voxels**
**(A) L-TLE vs. control**			
L Inferior frontal gyrus, opercular part	−45, 6, 12	0.53	32
L Middle temporal gyrus	−36, 6, −30	0.65	158
L Hippocampus	−30, −21, −9	0.66	294
L Caudate nucleus	−12, 18, 12	0.66	41
L Thalamus	−9, −18, 6	0.64	93
R Rolandic operculum	48, 6, 12	0.65	36
**(B) R-TLE vs. control**			
L Inferior frontal gyrus, triangular part	−51, 21, 15	0.70	218
L Superior frontal gyrus, medial	0, 33, 42	0.67	102
L Temporal pole: superior temporal gyrus	−36, 9, −30	0.61	43
L Anterior cingulate & paracingulate gyri	0, 39, 18	0.57	32
L Thalamus	−9, −15, 3	0.75	293
R Inferior frontal gyrus, triangular part	48, 18, 24	0.59	27

***(A)** The brain regions, whose GMD was related to the sum of the left thalamic subfield volume changes [left AV, LD, LP, CeM, CL, MV(Re)] in L-TLE*.

***(B)** The brain regions, whose GMD was related to the sum of the right thalamic subfield volume changes [right LD, MV(Re)] in L-TLE*.

*The results are listed below, and are also depicted in Figure 4*.

*The values given are the stereotactic MNI coordinates and the t-score of each anatomical region. SPM maps used a threshold of p < 0.01 (AlphaSim correction, voxel-level, minimum cluster size threshold of 19 voxels)*.

*Age and gender as covariates*.

*MNI: Montreal Neurological Institute*.

*L, left; R, right*.

The GMD changes in the ipsilateral side of the epileptogenic zone were more related to the volume changes of the thalamic subfields than the contralateral side in L-TLE. In contrast, R-TLE affected GMD more equally on both sides, but the effect was less extensive compared with the L-TLE. Most cortical volumes showed a positive relationship with the thalamic volumes, and only a few had a negative association in R-TLE (Table 7).

**Table 7:** The associated cortical area, whose GMD changes had the same degree of volumetric changes of the thalamic unit on each side in R-TLE.

**Regions**	**MNI coordinate**	**Peak *t*-score**	**Number of voxels**
**(A) L-TLE vs. control**			
L Precentral gyrus	−33, 0, 60	−0.65	19
R Inferior frontal gyrus, opercular part	51, 12, 33	−0.85	42
R Middle frontal gyrus	27, 48, 27	0.70	27
R Middle temporal gyrus	48, −51, 0	0.68	20
**(B) R-TLE vs. control**			
L Superior frontal gyrus, dorsolateral	−27, 54, 24	0.63	27
L Crus II of cerebellar hemisphere	−21, −72, −39	0.68	61
R Middle frontal gyrus	36, 36, 33	0.68	37
R Inferior frontal gyrus, opercular part	57, 15, 27	−0.69	21

***(A)** The brain regions, whose GMD was related to the sum of the left thalamic subfield volume changes (left LD, CL) in R-TLE*.

***(B)** The brain regions, whose GMD was related to the sum of the right thalamic subfield volume changes [right AV, LD, LP, VA, Vamc, CeM, MV(Re), MDl, L-Sg] in R-TLE*.

*The results are listed below, and are also depicted in Figure 5*.

*The values given are the stereotactic MNI coordinates and the t-score of each anatomical region. SPM maps used a threshold of p < 0.01 (AlphaSim correction, voxel-level, minimum cluster size threshold of 19 voxels)*.

*Age and gender as covariates*.

*MNI, Montreal Neurological Institute*.

*L, left; R, right*.

## Discussion

Our study used voxel- and volume-based morphometry to evaluate the extra-hippocampal gray matter volume changes in TLE, such as deep and superficial cortices. This study is the first to explore the relationship between volume changes of the deep and the superficial cortices to evaluate the reorganization of the hippocampo-thalamo-cortical volume in TLE using a volume-based morphometric method.

Our study found that the ipsilateral thalamic subfields had more prominent volume loss than those on the contralateral side, even though the whole thalamic volume did not obviously change. The previous literature has shown evidence of increased stereo-electroencephalography (SEEG) signal synchrony between the thalamus and the temporal lobe during the ictal event functionally (35). In addition, the functional study demonstrated that the thalamo-hippocampal connections were altered in pharmaco-resistant TLE (36). These findings confirmed that the subcortical gray matter also takes part in seizure circuits (10). The ratio of N-acetyl aspartate to creatine (NAA/Cr) measured by magnetic resonance spectroscopy (MRS) imaging was significantly correlated between the ipsilateral hippocampus and both sides of the anterior and posterior thalami (17).

This result echoed our study that disclosed bilateral thalami were structurally affected in unilateral temporal epilepsy. In the previous literatures, the most commonly reported thalamic subfields that are involved in TLE are the medial pulvinar [by SEEG (7) and resting-state fMRI (rs-fMRI) (20)], the anterior nucleus [by rs-fMRI (20)], and the midline nuclei (37). Their ipsilateral involvement was present in both TLE groups in our study, and we further defined the structures in more detail. The studies that used functional imaging, had the greatest spatial resolution of only about 3 mm due to the imaging limitation. Our study used structural imaging, which possessed a better spatial resolution to 1 mm. Therefore, we were able to explore the structures that were affected and changed in more detail, which allowed us to survey and identify the smaller structures that participated in the network reorganization.

Among the thalamic subfields with prominent volume loss, ipsilateral anteroventral nucleus was the most affected part of the thalamus in our study. Bilateral anterior thalamic nuclei atrophy has been observed in medial TLE, and the degree of volume loss was related to hippocampal CA1 volume (38). In one recent literature, it has been reported that high-frequency stimulation (130 Hz) of the thalamic anterior nucleus resulted in ipsilateral neural activity decoupling and desynchronization of the electrical background of the hippocampus (39). This resulted in the termination of seizure activity. However, lower-frequency stimulation (15–45 Hz) induced reciprocal thalamic-hippocampal evoked potentials. This phenomenon suggests the important role of anterior thalamic nuclei in the modulation of seizure activity. The anterior thalamic nucleus is a part of the Papez circuit, which is responsible for episodic memory (40), and it conducts reciprocal signals to the hippocampus, which is thought to maintain the seizure activity (41). Therefore, it is a common target for deep brain stimulation (DBS) in treating drug-resistant epilepsy (42). The anterior thalamic nuclei are subdivided into three parts based on their signal input and output: anteromedial, anteroventral, and anterodorsal (41). In our study, only the anteroventral subnucleus had a significantly decreased volume, with mutual connections to the subiculum cortex, which participates in the hippocampal circuit.

The ipsilateral central medial (CeM) and medial ventral (MVRe) nuclei showed significant atrophy in our study. The SEEG investigation found that the midline thalamic nuclei were involved in early recruitment in seizure initiation and propagation (36, 37). Therefore, their significant volume reduction on the side ipsilateral to the side affected by epilepsy was expected. Moreover, ipsilateral lateral nuclei showed prominent volume loss in our study. This phenomenon was observed in another study, which presented in TLE without hippocampal sclerosis (38). Both anterior and laterodorsal thalamic nuclei are the most important high-order relays to the cortex, having widespread links toward the frontal cortex and the hippocampal formation (43). Therefore, they are susceptible when the seizure originates in the hippocampus.

There is another recent study that FreeSurfer software was used to analyze the thalamic volume changes in TLE with hippocampal sclerosis (44). They found atrophic thalamic subfields were related to the type and the lateralization of hippocampal sclerosis, but the involved subfields were different from those identified in our present study. This might be because we enrolled only patients with pharmaco-resistant medial TLE, and we did not solely restrict enrollment to cases with hippocampal sclerosis. In addition, we adjusted our analyses for age and gender.

The duration of epilepsy has been reported to be related to changes in whole thalamic volume and connectivity (22, 45). The parameters of seizure burden showed a negative relationship with most of the thalamic subfield volumes in our study. These cumulative insults on the thalamic neurons resulted in neuronal death and then volume reduction. However, the seizure frequency of L-TLE was only positively related to the contralateral thalamic subfield volumes. This is thought to be a compensatory effect whereby the neurons in the contralateral thalamus might become hypertrophic to increase its volume. L-TLE was most susceptible to the long-term cumulative seizure burdens, exhibiting not only dramatically decreased bilateral whole thalamus volume but also decreases in the volumes of most individual subfields in cases with longer seizure duration. Thalamic volume in R-TLE seemed to be less affected by seizure burden.

Cortical thickness changes have been used to study the extratemporal cortical involvement in TLE in several previous studies, and the frontal, parietal, and occipital regions were all shown to be affected (25, 26, 46). Significant thinning was present over the bilateral precentral, paracentral, and frontal opercular gyri, as well as ipsilateral medial orbital cortex (26). Our study used voxel-based analysis of GMD to determine the cortical volume, instead of surface-based mapping using the indentation distance from the estimated surface (47). In contrast with previous studies, we considered the lateralization of TLE, age/gender correction, and added a stricter *p* (0.01 vs. 0.05) into the analytic method. Although we found the less extensive areas of GMD changes, these regions might be more significantly involved in the epilepsy network.

We found both R-TLE and L-TLE had a common pattern of cortical volume loss, which was in the ipsilateral precentral and the inferior frontal gyri. Precentral gyrus atrophy has been observed in TLE (26, 46), which was a characteristic used to differentiate cases of amnestic cognitive impairment from TLE (46). Primary motor cortex is located in the precentral gyrus, and was reported to reorganize in L-TLE, resulting in handedness shifting (48). However, an underlying network linking the thalamus or hippocampus to the precentral gyrus has been proposed, but has not yet been well confirmed (6). Therefore, further study on the network topology of the primary motor cortex in TLE is needed.

Inferior frontal gyri are the part of the prefrontal areas and consist of three parts: triangular, opercular, and orbital. Ipsilateral triangular part atrophy was prominent in both TLE groups when compared with controls. However, GMD changes of the ipsilateral opercular part were related to the sum of volume loss of the thalamic subfields. Both triangular and opercular parts belong to the Broca's area in the dominant hemisphere. Frontal dysfunction has been observed in TLE, as demonstrated in neuropsychological and neuroimaging studies (49). Disruption of thalamocortical connections has been proposed to cause impaired executive and attention function in TLE, which suggested that the frontal networks were involved (50). One MRI study presented impaired dorsolateral prefrontal cortex—thalamus connectivity in pharmaco-resistant TLE using diffusion tensor imaging (51). Therefore, our study used the higher-resolution imaging to confirm the relationship between the prefrontal cortex and the anterior thalamus structurally. Other parts of the prefrontal cortex, such as the middle and superior frontal gyri, were shown to exhibit GMD changes that were significantly related to the volume changes of the thalamic subfields in our results. However, some of these changes were found to have a negative relationship, possibly due to hypertrophic neurons (24), and predominantly occurring in our R-TLE. Therefore, we propose that prefrontal-thalamic-hippocampal networks are affected in both sides of TLE because they presented synchronously volumetric changes. Moreover, basal ganglion and cerebellum volume changes were found, and their thalamic connections in TLE need further exploration.

The limitations of our studies were the limited sample size, and we did not group TLE according to the presence of hippocampal sclerosis or not. Furthermore, the neuropsychological scores and functional neuroimaging were not simultaneously evaluated in our subjects. Therefore, it was difficult to interpret the effect of volume on their cognitive functions.

## Conclusion

We obtained more detailed structural information related to disrupted thalamo-temporal networks based on the volumetric changes of the subfields in the thalamus. Ipsilateral anteroventral nucleus was the most susceptible subfield in both the left and right TLE, and exhibited most atrophy. Most thalamic subfields were susceptible to long-term seizure burden, especially L-TLE. The involved cortical regions with decreased GMD in TLE were beyond the temporal cortices. Ipsilateral precentral and inferior frontal gyri were prominently affected. The GMD changes of the ipsilateral inferior frontal gyri were positively or negatively related to the sum of volume changes of the each side of thalamic subfields, which suggests the alteration and disruption of prefrontal-thalamic-hippocampal volumetric reorganization in TLE.

## Data Availability Statement

The raw data supporting the conclusions of this article will be made available by the authors, without undue reservation.

## Ethics Statement

The studies involving human participants were reviewed and approved by the Ethics Committee of Taichung Veterans General Hospital, Taichung, Taiwan (CE18306B). Written informed consent for participation was not required for this study in accordance with the national legislation and the institutional requirements.

## Author Contributions

HT and S-JP designed and analyzed the data, drafted the manuscript, and edited the manuscript. S-JP analyzed the data. S-YP, T-HL, and Y-YL contributed to revising the manuscript. All authors contributed to the article and approved the submitted version.

## Funding

This study was supported by a grant from the Ministry of Science and Technology, Taiwan, under the project MOST 110-2221-E-038-008.

## Conflict of Interest

The authors declare that the research was conducted in the absence of any commercial or financial relationships that could be construed as a potential conflict of interest.

## Publisher's Note

All claims expressed in this article are solely those of the authors and do not necessarily represent those of their affiliated organizations, or those of the publisher, the editors and the reviewers. Any product that may be evaluated in this article, or claim that may be made by its manufacturer, is not guaranteed or endorsed by the publisher.
