# Supplementary material for: Characterization of Hippocampal-Thalamic-Cortical Morphometric Reorganization in Temporal Lobe Epilepsy
Source: Front Neurol. 2022 Feb 10;12:810186. doi: 10.3389/fneur.2021.810186 (PMC8866816; doi:10.3389/fneur.2021.810186)
Supplement: Supplementary file 1 [file Table_1.docx]

**Supplementary Table 1. The detail information of the epilepsy patients.**

| **code** | **hippocampal sclerosis**  **(0=no, 1=yes)** | **Gender**  **(0=male, 1=female)** | **Age** | **AED number** | **Seizure duration since diagnosis**  **(years)** | **Seizure frequency**  **(per month)** | **spikes in routine EEG**  **(0=yes, 1=no)** |
| --- | --- | --- | --- | --- | --- | --- | --- |
| R-TLE-01 | 0 | 0 | 27 | 1 | 1 | 0.5 | 1 |
| R-TLE-02 | 0 | 1 | 36 | 2 | 3 | 0.5 | 1 |
| R-TLE-03 | 0 | 1 | 49 | 2 | 5 | 1 | 1 |
| R-TLE-04 | 0 | 0 | 54 | 5 | 30 | 3 | 0 |
| R-TLE-05 | 1 | 1 | 65 | 3 | 30 | 2 | 1 |
| R-TLE-06 | 1 | 0 | 29 | 4 | 17 | 2 | 0 |
| R-TLE-07 | 1 | 1 | 46 | 3 | 17 | 0 | 0 |
| R-TLE-08 | 1 | 0 | 27 | 4 | 9 | 2 | 0 |
| R-TLE-09 | 1 | 0 | 53 | 5 | 20 | 3 | 1 |
| R-TLE-10 | 0 | 0 | 39 | 1 | 1 | 0 | 0 |
| R-TLE-11 | 0 | 1 | 39 | 2 | 2 | 0 | 0 |
| R-TLE-12 | 0 | 1 | 33 | 4 | 9 | 6 | 1 |
| R-TLE-13 | 0 | 1 | 51 | 3 | 28 | 3 | 1 |
| R-TLE-14 | 0 | 0 | 22 | 2 | 1 | 1 | 0 |
| R-TLE-15 | 0 | 0 | 56 | 1 | 1 | 0.5 | 0 |
| R-TLE-16 | 0 | 1 | 63 | 1 | 20 | 3 | 0 |
| R-TLE-17 | 1 | 1 | 66 | 3 | 30 | 2 | 1 |
| R-TLE-18 | 1 | 0 | 69 | 2 | 25 | 0.5 | 0 |
| R-TLE-19 | 0 | 1 | 37 | 1 | 8 | 2 | 0 |
| R-TLE-20 | 1 | 0 | 29 | 2 | 26 | 5 | 1 |
| R-TLE-21 | 0 | 1 | 37 | 1 | 3 | 1 | 0 |
| R-TLE-22 | 0 | 0 | 24 | 3 | 17 | 3 | 1 |
| L-TLE-01 | 1 | 0 | 54 | 2 | 20 | 1 | 0 |
| L-TLE-02 | 1 | 0 | 38 | 3 | 28 | 4 | 0 |
| L-TLE-03 | 1 | 0 | 27 | 2 | 9 | 3 | 0 |
| L-TLE-04 | 0 | 1 | 22 | 2 | 2 | 0 | 0 |
| L-TLE-05 | 1 | 0 | 33 | 5 | 23 | 3 | 0 |
| L-TLE-06 | 1 | 0 | 51 | 4 | 11 | 4 | 1 |
| L-TLE-07 | 0 | 0 | 38 | 4 | 31 | 0.5 | 1 |
| L-TLE-08 | 0 | 1 | 19 | 3 | 2 | 4 | 1 |
| L-TLE-09 | 0 | 0 | 24 | 3 | 2 | 0.5 | 1 |
| L-TLE-10 | 0 | 0 | 34 | 2 | 5 | 8 | 1 |
| L-TLE-11 | 1 | 1 | 29 | 2 | 5 | 0 | 0 |
| L-TLE-12 | 1 | 0 | 55 | 1 | 8 | 2 | 1 |
| L-TLE-13 | 1 | 1 | 41 | 4 | 28 | 3 | 1 |
| L-TLE-14 | 0 | 1 | 31 | 3 | 22 | 2 | 1 |
| L-TLE-15 | 0 | 1 | 65 | 1 | 2 | 1 | 1 |
| L-TLE-16 | 1 | 1 | 63 | 2 | 63 | 0.5 | 1 |
| L-TLE-17 | 0 | 0 | 26 | 2 | 6 | 0.5 | 1 |
| L-TLE-18 | 0 | 0 | 24 | 2 | 2 | 0 | 0 |
| L-TLE-19 | 0 | 0 | 54 | 2 | 1 | 1 | 0 |
| L-TLE-20 | 0 | 0 | 32 | 2 | 3 | 0.5 | 0 |
| L-TLE-21 | 1 | 1 | 47 | 2 | 44 | 2 | 1 |
| L-TLE-22 | 1 | 1 | 33 | 2 | 14 | 4 | 1 |
| L-TLE-23 | 1 | 0 | 23 | 3 | 17 | 2 | 1 |
| L-TLE-24 | 1 | 0 | 50 | 3 | 10 | 3 | 1 |
| L-TLE-25 | 0 | 1 | 35 | 4 | 16 | 6 | 0 |
| L-TLE-26 | 1 | 0 | 31 | 2 | 4 | 2 | 1 |
| L-TLE-27 | 0 | 0 | 19 | 2 | 1 | 4 | 1 |
| L-TLE-28 | 1 | 1 | 31 | 3 | 17 | 2 | 0 |
| L-TLE-29 | 0 | 0 | 41 | 3 | 2 | 0.5 | 0 |
| L-TLE-30 | 0 | 0 | 56 | 1 | 1 | 1 | 0 |
| L-TLE-31 | 0 | 1 | 51 | 1 | 2 | 0.5 | 0 |
| L-TLE-32 | 1 | 1 | 31 | 4 | 21 | 2 | 1 |
| L-TLE-33 | 0 | 0 | 42 | 2 | 17 | 0.5 | 0 |
